# A Comparative Study of Split-Thickness Skin Graft Fixation and Uptake Using Autologous Platelet-Rich Plasma Versus Conventional Methods

**DOI:** 10.7759/cureus.85136

**Published:** 2025-05-31

**Authors:** Divyang GB, Manjunath Kotennavar, Aravind V Patil, Pradeep P Jaju, Sanjeev Rathod, Manjunath S Savant, Veena Ghanteppagol, Shreeya Doddannavar, Eswar Medikonda, Smit Parikh

**Affiliations:** 1 Surgery, Shri BM Patil Medical College, Hospital and Research Centre, BLDE (Deemed to be University), Vijayapura, IND; 2 General Surgery, Shri BM Patil Medical College, Hospital and Research Centre, BLDE (Deemed to be University), Vijayapura, IND

**Keywords:** autologous platelet-rich plasma, graft adhesion, graft fixation, graft uptake, reconstruction, skin defects, split-thickness skin graft, wound healing

## Abstract

Introduction: Split-thickness skin grafting is a cornerstone in reconstructive surgery for managing skin defects. However, graft failure remains a significant challenge. This study evaluated the effectiveness of autologous platelet-rich plasma (PRP) in enhancing split-thickness skin graft (STSG) fixation and uptake compared to conventional methods.

Methods: This prospective comparative study included 104 patients requiring STSGs, randomly allocated into two equal groups (n=52 (50%) each). The PRP group received autologous PRP application at the recipient site before graft placement, while the control group underwent conventional fixation with staples/sutures. Outcomes were assessed based on immediate postoperative adhesion, graft uptake percentage, and complications (edema and hematoma) at days 3, 5, and 7 postoperatively.

Results: The groups were comparable in terms of demographic characteristics and wound parameters. Immediate postoperative adhesion was observed in 36 (69.2%) patients in the PRP group versus none in the control group (p<0.001). The PRP group demonstrated significantly higher graft uptake on day 3 (96.07±5.7% versus 86.33±6.06%, p<0.001) and day 5 (92.7±8.4% versus 79.9±9.1%, p<0.001), with sustained difference on day 7 (90.8±10.5% versus 75.62±11.2%, p=0.07). The PRP group exhibited significantly less graft edema and hematoma throughout the follow-up period (p<0.001).

Conclusion: Autologous PRP significantly enhances STSG fixation and uptake, improves overall graft take rates, and reduces postoperative complications. The immediate adhesion effect provides enhanced graft stability without additional mechanical fixation, which is valuable in anatomically challenging areas. The improvement in graft take rate is clinically significant and can translate into reduced need for regrafting, shorter hospital stays, and improved outcomes. PRP's anti-inflammatory, pro-angiogenic, and hemostatic properties create an optimal environment for graft integration, supporting its incorporation as a valuable adjunct in split-thickness skin grafting procedures.

## Introduction

Skin grafting represents one of the oldest and most fundamental techniques in reconstructive surgery, with its first documented use dating back to ancient Indian civilization. The procedure has evolved significantly over centuries, yet remains a cornerstone in managing various soft tissue defects, whether from trauma, burns, chronic wounds, or surgical resections [1,2]. In particular, split-thickness skin grafts (STSGs) have emerged as a versatile and reliable option in reconstructive surgery, offering both functional and aesthetic restoration of skin defects [3].

The success of skin grafting procedures fundamentally depends on graft survival, which is influenced by multiple factors, including the quality of the recipient bed, graft fixation techniques, and the complex biological processes that occur during graft take. Traditional methods of STSG fixation, including sutures, staples, and various dressing techniques, have shown varying degrees of success, with reported graft take rates ranging from 70% to 90%, depending on the location and condition of the recipient site [4]. However, these conventional approaches often face challenges such as graft displacement, seroma formation, and incomplete graft-to-bed contact, which can compromise graft survival.

In recent years, there has been growing interest in the application of autologous platelet-rich plasma (PRP) in various aspects of wound healing and tissue regeneration. PRP, defined as an autologous concentration of platelets in a small volume of plasma, contains numerous growth factors and bioactive proteins that are essential for tissue repair and regeneration [4,5]. These include platelet-derived growth factor (PDGF), transforming growth factor-β (TGF-β), vascular endothelial growth factor (VEGF), and epidermal growth factor (EGF), among others [6]. The theoretical basis for using PRP in skin grafting lies in its potential to enhance the graft's initial adhesion through its high fibrin content and the subsequent revascularization process through its rich array of growth factors [7].

Therefore, the aim of this study was to compare STSG fixation and uptake using autologous PRP versus conventional methods.

## Materials and methods

This prospective comparative study was conducted in the Department of Surgery at BLDE University’s Shri BM Patil Medical College, Hospital and Research Centre, Vijayapura, between April 2023 and March 2025. The sample size of 104 (52 in each group) was calculated using G*Power software (Heinrich-Heine-Universität Düsseldorf, Düsseldorf, Germany), targeting a 97% power and 1% significance level to detect differences in graft edema proportions between groups. A total of 104 patients requiring split-thickness skin grafting for ulcers of various etiologies were included and allocated into two equal groups: the study group, where autologous PRP was used for graft fixation, and the control group, where conventional methods such as staples or sutures were used.

Patients aged between 18 and 75 years with ulcers of any etiology and size less than 15 × 15 cm were included in the study. Exclusion criteria were the presence of skin malignancies, critically ill patients, and those with known platelet disorders. All patients underwent appropriate wound bed preparation before surgery. STSGs were harvested using standard techniques with a Humby’s knife. In the study group, PRP was prepared from the patient's venous blood using a double centrifugation method and applied directly to the wound bed before graft placement. In the control group, grafts were secured using conventional staples or sutures without PRP application.

**Figure 1:**
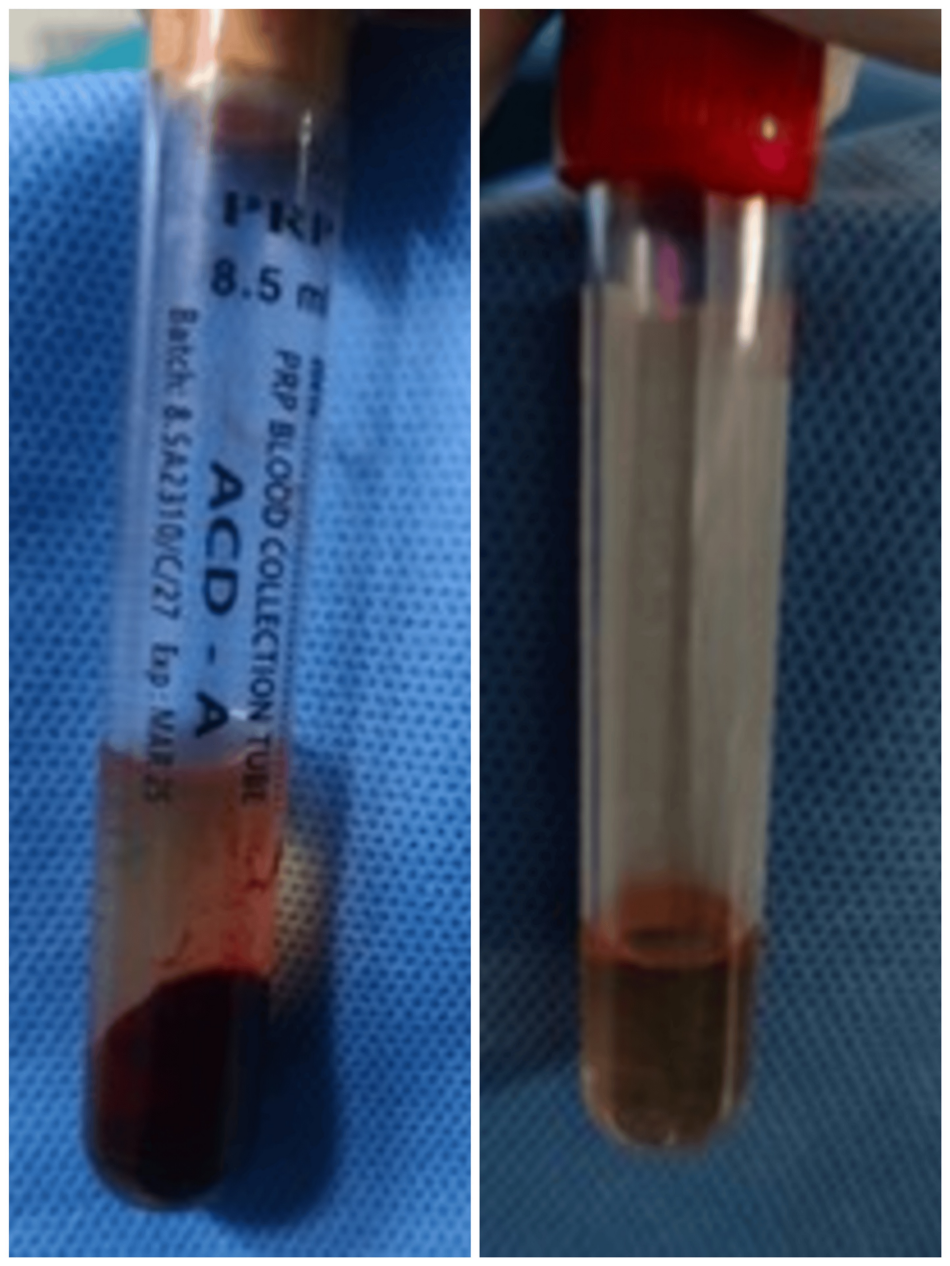
Preparation of PRP PRP was prepared using a double centrifugation method, initially in ACD-A vacutainers, followed by transfer and final spin in plain vacutainers to concentrate platelets. PRP: Platelet-rich plasma; ACD-A: Acid citrate dextrose-A

**Figure 2:**
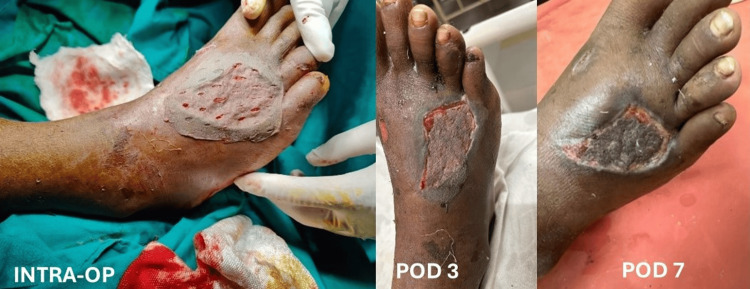
Outcome of PRP application Intraoperative and postoperative images showing fixation of STSG with the aid of autologous PRP, demonstrating adhesion and healing. PRP: Platelet-rich plasma; STSG: Split-thickness skin graft; POD: Postoperative day; INTRA-OP: Intraoperative

Postoperative outcomes were evaluated based on immediate graft adhesion, percentage of graft uptake, and complications such as graft edema and hematoma. These parameters were assessed on postoperative days 3, 5, and 7. Statistical analysis was performed using IBM SPSS Statistics Version 26 (IBM Corp., Armonk, US). A p-value of less than 0.05 was considered statistically significant. Appropriate statistical tests, including the independent sample t-test and chi-squared test, were applied. The study was approved by the Institutional Ethics Committee, reference number BLDE (DU)/IEC/921/2023-24 (Figures 1-2).

## Results

The demographic and wound characteristics of the study participants were comparable between the two groups. The majority of patients belonged to the 41-80 year age range in both the PRP and control groups, with no statistically significant difference in age distribution (p=0.3636). Male predominance was observed in both groups, though the gender distribution did not differ significantly (p=0.6838). The mean ulcer size was slightly larger in the PRP group (8.3±3.6 cm²) compared to the control group (7.9±3.8 cm²), but this difference was not statistically significant (p=0.57). The upper limb was the most common site for ulcers in both groups, followed by the lower limb and back, with no significant variation in ulcer site distribution (p=0.4997) (Table 1).

**Table 1:** Demographic and wound characteristics of study participants Categorical variables are expressed as frequencies and percentages, while continuous variables are expressed as mean ± standard deviation (SD). A p-value of less than 0.05 is considered statistically significant. PRP: Platelet-rich plasma

Characteristics		PRP group (n=52)	Control group (n=52)	Statistical value	p-value
Age (years)	<20	3 (2.8%)	2 (1.9%)	χ² = 4.3262	0.3636
	20-40	6 (5.7%)	14 (13.4%)		
	41-60	21 (20.1%)	17 (16.3%)		
	61-80	20 (19.2%)	15 (14.4%)		
	>80	2 (1.9%)	4 (3.8%)		
Gender	Female	20 (19.2%)	18 (17.3%)	χ² = 0.1658	0.6838
	Male	32 (30.7%)	34 (32.6%)		
Ulcer size (cm²)		8.3±3.6	7.9±3.8	t = 0.57	0.57
Ulcer site	Upper limb	36 (34.6%)	38 (36.5%)	χ² = 1.3873	0.4997
	Lower limb	14 (13.4%)	10 (9.6%)		
	Back	2 (1.9%)	4 (3.8%)		

Regarding the etiology of ulcers, necrotizing fasciitis and diabetic foot ulcers were the most common causes in both groups. Necrotizing fasciitis accounted for 24 cases in the PRP group and 20 in the control group, while diabetic foot ulcers were slightly more prevalent in the control group (26 cases) than in the PRP group (20 cases). Burns and trauma were relatively uncommon in both groups. However, the differences in etiology between the groups were not statistically significant (p=0.2823) (Table 2). 

**Table 2:** Etiology of ulcers in both groups Categorical variables are expressed as frequencies and percentages. A p-value of less than 0.05 is considered statistically significant. PRP: Platelet-rich plasma

Etiology	PRP group (n=52)	Control group (n=52)	Statistical value	p-value
Necrotizing fasciitis	24 (23%)	20 (19.2%)	χ² = 3.8129	0.2823
Diabetic foot ulcer	20 (19.2%)	26 (25%)		
Burns	2 (1.9%)	4 (3.8%)		
Trauma	6 (5.7%)	2 (1.9%)		

A marked difference was observed in immediate postoperative graft adhesion, with 69.2% of patients in the PRP group exhibiting successful adhesion compared to none in the control group - a difference that was highly significant (p<0.001). Graft uptake was significantly better in the PRP group on postoperative days 3 and 5 (p<0.001 for both), while the difference on day 7 was substantial but did not reach statistical significance (p=0.07) (Table 3).

**Table 3:** Immediate postoperative adhesion and graft uptake at different time points Categorical variables are expressed as frequencies and percentages, while continuous variables are expressed as mean ± standard deviation (SD). A p-value of less than 0.05 is considered statistically significant. PRP: Platelet-rich plasma

Parameter		PRP group (n=52)	Control group (n=52)	Statistical value	p-value
Immediate adhesion		36 (69.2%)	0 (0%)	χ² = 44.1290	<0.001
Graft uptake (%)	Day 3	96.07±5.7	86.33±6.06	t = 9.01	<0.001
	Day 5	92.7±8.4	79.9±9.1	t = 7.74	<0.001
	Day 7	90.8±10.5	75.62±11.2	t = 1.86	0.07

Graft edema was significantly lower in the PRP group across all postoperative days assessed. On day 3, minimal edema was seen in 32 (61.5%) PRP patients versus 13 (25%) in the control group (p=0.0005). By day 5, 42 (80.7%) PRP patients had minimal edema compared to 23 (44.2%) in the control group (p=0.0001), and on day 7, 47 (90.3%) in the PRP group had minimal edema versus 35 (67.3%) in the control group (p=0.0039), showing consistent superiority of PRP in reducing graft edema (Table 4). 

**Table 4:** Graft edema at different time points Categorical variables are expressed as frequencies and percentages. A p-value of less than 0.05 is considered statistically significant. PRP: Platelet-rich plasma

Graft edema	Time point	PRP group (n=52)	Control group (n=52)	Statistical value	p-value
Day 3	Minimal	32 (61.5%)	13 (25%)	χ² = 14.921	0.0005
	Moderate	17 (32.6%)	29 (55.7%)		
	Gross	3 (5.7%)	10 (19.3%)		
Day 5	Minimal	42 (80.7%)	23 (44.2%)	χ² = 14.8102	0.0001
	Moderate	10 (19.2%)	29 (55.7%)		
Day 7	Minimal	47 (90.3%)	35 (67.3%)	χ² = 8.3015	0.0039
	Moderate	5 (9.6%)	17 (32.6%)		

Similarly, the incidence of hematoma was significantly lower in the PRP group at all time points. On day 3, 46 (88.4%) PRP patients had minimal hematoma compared to only 17 (32.6%) in the control group (p<0.001). This trend persisted on day 5 (50 (96.1%) versus 31 (59.6%), p<0.001) and day 7 (50 (96.1%) versus 38 (73%), p<0.001), indicating better hemostatic control with PRP application (Table 5).

**Table 5:** Hematoma at different time points Categorical variables are expressed as frequencies and percentages. A p-value of less than 0.05 is considered statistically significant. PRP: Platelet-rich plasma

Hematoma	Time point	PRP group (n=52)	Control group (n=52)	Statistical value	p-value
Day 3	Minimal	46 (88.4%)	17 (32.6%)	χ² = 33.8614	<0.001
	Moderate	6 (11.5%)	35 (67.3%)		
Day 5	Minimal	50 (96.1%)	31 (59.6%)	χ² = 20.1524	<0.001
	Moderate	2 (3.8%)	21 (40.3%)		
Day 7	Minimal	50 (96.1%)	38 (73%)	χ² = 20.4565	<0.001
	Moderate	2 (3.8%)	14 (26.9%)		

## Discussion

The application of autologous PRP in split-thickness skin grafting demonstrated significant advantages over conventional fixation methods in this study. The most striking finding was the immediate postoperative adhesion observed in 36 (69.2%) patients in the PRP group, whereas none in the conventional group exhibited this phenomenon (p<0.001). This instant adhesion can be attributed to the fibrin-rich nature of PRP, which provides an immediate biological adhesive effect. Kakudo et al. [8] demonstrated similar findings, reporting enhanced adherence of skin grafts when PRP was applied to the wound bed before graft placement. They proposed that the fibrin in PRP helps in securing the graft to the wound bed, minimizing micro-motion, which is detrimental to graft take. This immediate adhesion is particularly beneficial in anatomically challenging regions where conventional dressings might not provide optimal graft-recipient contact.

The superior graft uptake percentages observed in the PRP group (96.07% versus 86.33% on day 3, 92.7% versus 79.9% on day 5, and 90.8% versus 75.62% on day 7) highlight the sustained beneficial effects of PRP on graft survival. This enhanced graft take can be attributed to multiple mechanisms through which PRP affects wound healing. Growth factors such as VEGF and fibroblast growth factor (FGF) in PRP promote angiogenesis, facilitating earlier revascularization of the graft [9]. This ensures adequate nutrition and oxygenation, critical for graft survival. Additionally, growth factors such as PDGF and EGF stimulate the proliferation and migration of fibroblasts, keratinocytes, and endothelial cells, accelerating wound healing and graft integration [10]. The TGF-β present in PRP has also been associated with reduced scar formation, potentially leading to better aesthetic outcomes. Our findings align with several studies in the literature that have reported enhanced graft take rates and reduced healing time with PRP application. Maghsoudi et al. [11] conducted a similar study focusing on diabetic foot ulcers and reported enhanced graft take rates and reduced healing time with PRP application.

The significantly reduced incidence of complications, including graft edema and hematoma formation, in the PRP group underscores the protective effects of PRP against factors that typically compromise graft survival. On postoperative day 3, 32 (61.5%) PRP group patients showed minimal edema compared to only 13 (25%) in the conventional group (p<0.001), and 46 (88.4%) showed minimal hematoma compared to 17 (32.6%) in the control group (p<0.001). This trend persisted throughout the follow-up period. The reduced incidence of these complications in the PRP group can be attributed to the hemostatic and anti-inflammatory properties of PRP. The concentrated platelets in PRP release thromboxane A2, which promotes vasoconstriction and platelet aggregation, thus minimizing bleeding and subsequent hematoma formation [12]. PRP also contains anti-inflammatory cytokines that modulate the inflammatory response in the wound bed. Excessive inflammation can damage the graft, and its modulation contributes to improved graft survival. These multifaceted mechanisms work synergistically to enhance graft take and minimize complications. Venter et al. [13] and Pallua et al. [14] similarly reported reduced complications and enhanced graft take rates with PRP application, even at the end of the first week post-surgery.

Despite the encouraging outcomes observed in this study, several limitations must be acknowledged. The sample size, although adequate to show statistical significance in primary outcomes, limits the generalizability of the results across broader populations and diverse clinical scenarios. The study was conducted in a single tertiary care center, which may introduce center-specific biases related to surgical technique, postoperative care, and patient characteristics. Additionally, the follow-up period was limited to the immediate postoperative phase (up to day 7), preventing assessment of long-term outcomes such as graft durability, scarring, and patient satisfaction. Variability in ulcer etiology, though balanced between groups, may still influence individual healing responses, which were not fully stratified in subgroup analysis. Finally, the preparation and application of PRP were not standardized across all settings, and inter-individual variation in platelet concentration may have affected the outcomes. Future multicenter studies with larger sample sizes and longer follow-up durations are warranted to validate and extend these findings.

## Conclusions

This prospective comparative study demonstrated that autologous platelet-rich plasma significantly enhances STSG fixation and uptake compared to conventional methods. The findings of this study support the incorporation of autologous PRP as a valuable adjunct in split-thickness skin grafting procedures. Being autologous in nature, PRP offers these benefits without the risks associated with allogenic or synthetic products. The technique is relatively simple, cost-effective, and can be easily integrated into existing surgical protocols without significant additional resources.
